# Supplementary material for: Rapid turnover of life-cycle-related genes in the brown algae
Source: Genome Biol. 2019 Feb 14;20:35. doi: 10.1186/s13059-019-1630-6 (PMC6374913; doi:10.1186/s13059-019-1630-6)
Supplement: Supplementary file 3 — Protein alignments, corresponding Gblocks html file and CDS sequences for the lineage conserved gametophyte-biased genes. (ZIP 2109 kb) [file 13059_2019_1630_MOESM3_ESM.zip › Con_GA_alignments/SJ14991.aa_ali.fasta-gb.htm]

SJ14991.aa.fasta.trans.aa\_ali.fasta


## Gblocks 0.91b Results

Processed file: **SJ14991.aa.fasta.trans.aa\_ali.fasta**  
Number of sequences: **2**  
Alignment assumed to be: **Protein**  
New number of positions: **157** (selected positions are underlined in blue)

```
                         10        20        30        40        50        60
                 =========+=========+=========+=========+=========+=========+
SJ14991          MGTTTTTTRRMGYEYDSHDHSRGSGSGVVPVRRPTRLRGRVDSAKAEELVAGLGFNVAGS
Mac.30566.1      ------------------------------------------------------------
                                                                             


                         70        80        90       100       110       120
                 =========+=========+=========+=========+=========+=========+
SJ14991          TTSSPSSSTSSSSTSSTPDVRKGRRRRRADSAAPTPAGGGDDDDDQENLRFLEAADFTVP
Mac.30566.1      ------------------------------------------------------------
                                                                             


                        130       140       150       160       170       180
                 =========+=========+=========+=========+=========+=========+
SJ14991          VEDRLAFERNGHTVTRGVFSEGQLKALRPSIVRAFERQEMEALRQKVSVWFGDETASGLE
Mac.30566.1      ------------------------------------------------------------
                                                                             


                        190       200       210       220       230       240
                 =========+=========+=========+=========+=========+=========+
SJ14991          TAPELRARLKRLAPEEIPFLQVFNMFRKAGPYREAVEKVVLSPRLAHVAAQLLGCERVKL
Mac.30566.1      ------------------------MFRKAGPYQEAVEKVVLSPRLAHVAAQLLGCERVKL
                                         ####################################


                        250       260       270       280       290       300
                 =========+=========+=========+=========+=========+=========+
SJ14991          YQDSTFCKRPGDGPTRWHSDLNMAPLDCNDFVTAWIPLEPVPARKEGGSPLVFASRSHRD
Mac.30566.1      YQDSTFCKRPGDGPTRWHSDLNMAPLDCNDFVTAWIPLEPVPARKKGGSPLVFASRSHRD
                 ############################################################


                        310       320       330       340       350       360
                 =========+=========+=========+=========+=========+=========+
SJ14991          FALNHWLDAKLLKDLSGRYREKDHAPLSAGDITWHHGWTLHSSPGNARDEPRTAFAISYL
Mac.30566.1      FALNHWLDAKLQEDLSGRYKKKDHAPLSAG-------------------EPVMAIASPSS
                 ##############################                   ###########


                        370       380       390       400       410
                 =========+=========+=========+=========+=========+=====
SJ14991          RDGTRLLSDSTGRKPDPEDKWSYGDWAKELPPGGVIEHPSVPLVWNGGKPLSSKR
Mac.30566.1      RGVHQLFDAMVDTHCVPYEYISK--------PSLSQSHPASPVAW----------
                 ######                         ##############
```

```
Parameters used
Minimum Number Of Sequences For A Conserved Position: 2
Minimum Number Of Sequences For A Flanking Position: 2
Maximum Number Of Contiguous Nonconserved Positions: 8
Minimum Length Of A Block: 10
Allowed Gap Positions: None
Use Similarity Matrices: Yes
```

```
Flank positions of the 3 selected block(s)
Flanks: [205  330]  [350  366]  [392  405]  

New number of positions in SJ14991.aa.fasta.trans.aa_ali.fasta-gb:  157  (37% of the original 415 positions)
```
